# Enhanced external counter pulsation in treatment of refractory angina pectoris: two year outcome and baseline factors associated with treatment failure

**DOI:** 10.1186/1471-2261-8-39

**Published:** 2008-12-18

**Authors:** André Erdling, Susanne Bondesson, Thomas Pettersson, Lars Edvinsson

**Affiliations:** 1 Department of Medicine, Centralsjukhuset, SE-291 85 Kristianstad, Sweden; 2 Department of Emergency Medicine, Clinical Sciences Lund, Lund University, SE-221 85 Lund, Sweden

## Abstract

**Background:**

Enhanced external counter pulsation (EECP) is a non-invasive treatment option for patients with refractory angina pectoris ineligible to further traditional treatment. The aim of this study was to evaluate the effect of EECP on patients at a Scandinavian medical centre and to investigate if outcome can be predicted by analysing baseline factors.

**Methods:**

86 consecutive patients (70 male, 16 female) were treated with EECP and followed for two years post treatment. Canadian cardiovascular society (CCS) class was analysed, and medication and adverse clinical events were researched prior to EECP, at the end of the treatment, and at six, 12 and 24 months thereafter. Patients responding to therapy by improving at least one CCS class were compared with those who failed to respond. Any differences in background factors were recorded and analysed.

**Results:**

79% of the patients responded to therapy by improving at least one CCS class. In general, the CCS class improved by one class after EECP treatment (3.05 before versus 2.14 after treatment). A total of 61.5% of the initial responders showed sustained improvement at the 12 month follow-up while 29% presented sustained improvement after 24 months. Treatment was most effective among patients suffering from CCS class III-IV angina pectoris, while patients suffering from CCS class II angina pectoris improved transiently but failed to show sustained improvement after the 12 month follow-up. Diabetes mellitus and calcium channel antagonists were more common among the non-responders (*p *< 0.05).

**Conclusion:**

This study confirms the safety and efficiency of EECP as a treatment option for patients suffering from refractory angina pectoris. The therapy is most beneficial in patients suffering from severe angina (CCS III-IV) while sustained response to therapy could not be verified among patients suffering from CCS class II angina pectoris.

## Background

Stable angina pectoris is a common and sometimes disabling disorder characterized by chest pain due to ischemia of the myocardium, generally caused by obstruction or spasm of the coronary arteries. Atherosclerosis of coronary vessels is the main cause of angina pectoris while coronary artery spasm is seen in a minor number of angina pectoris patients. Other causes such as connective tissue disorders, valvular disease and arrhythmia are even less common.

Prevalence of angina pectoris increases with age in both males and females. It has been estimated that 2–4% of the adult European population is affected by angina pectoris [1]. This number is likely to increase further since life expectancy continues to improve worldwide.

Treatment of angina pectoris is traditionally aimed at reduction of symptoms as well as prevention of future cardiac events such as myocardial infarction or death. Pharmacological agents such as nitrates, aspirin, beta-adrenoreceptor antagonists and calcium channel blockers are used [2] as well as surgical therapies aimed at restoring blood flow, e.g. coronary artery bypass graft (CABG) or percutaneous coronary intervention (PCI) [3]. As many as 15% of the patients either fail to respond fully to therapy as described above or are ineligible to further intervention, thus said to suffer from refractory angina pectoris [4]. These patients suffer from marked limitation of everyday physical activity due to their pain, which in some cases are more or less constant. In the last few years, the lack of efficient therapy for refractory angina pectoris in combination with increased survival rates after myocardial infarction and an ageing population has caused increased need for new therapeutic methods. Intense research has yielded methods such as laser revascularization, left stellate ganglion blockade, spinal cord stimulation (SCS) and enhanced external counterpulsation (EECP)[3].

EECP is a non-invasive method used to treat patients with refractory angina pectoris, ineligible to further pharmacological or surgical intervention. Pneumatic cuffs are applied to the lower limbs and set to inflate sequentially during diastole and deflate before the onset of systole. This causes an increased diastolic pressure resulting in augmented coronary blood flow [5] as well as increased venous return [6] and improved cardiac output [7]. The systolic blood pressure is lowered due to deflation before systole, thereby decreasing afterload and preventing heart failure and pulmonary oedema [8].

The aim of the current study was to assess the two-year outcome of EECP treatment of refractory angina pectoris and to determine whether the response to treatment can be predicted by analysis of baseline factors.

## Methods

### Patients

A total of 86 consecutive patients (70 male, 16 female) were treated with EECP and followed for two years after completion of treatment.

Referral, treatment and follow-ups were conducted at the Central hospital in Kristianstad in southern Sweden. All patients had angiographically verified significant stenosis in at least one major coronary artery. Patients were considered at optimal pharmacological treatment and unsuitable for further revascularization by the time of referral as evaluated by a board of cardiologists and thoracic surgeons at the Lund University hospital. Anginal status, medical history, glyceryl trinitrate (GTN) consumption and demographics were obtained at baseline with follow-up immediately after treatment, at 6, 12 and 24 months after therapy. Pharmacological treatment was adjusted whenever the need for adjustments arose.

Patients were divided into two groups depending on response to therapy. Those who improved at least one Canadian cardiovascular society (CCS) class after treatment were considered responders whereas those who failed to respond to therapy were considered non-responders at the immediate follow-up. Baseline factors (Table 1) were compared between the two groups in an attempt to find factors predisposing patients to treatment failure. Patients had a long history of coronary artery disease (mean duration 11.5 years) and risk factors for arteriosclerosis were common. Most patients had suffered from one or more acute myocardial infarctions, most of which had been treated with either PCI or CABG.

**Table 1:** Baseline characteristics

	Total, n = 78	Responders, n = 62	Non-responders, n = 16	***p***
Gender	63M/15F	49M/13F	14M/2F	0.72
Age at referral	66, 43–87	64, 43–87	71, 55–84	0.14
Beta-adrenoceptor antagonists	57	45	12	1.00
Ca^2+^-channel antagonists	35	24	11	< 0.05
Long-lasting nitrates	58	45	15	0.10
Warfarin	6	4	2	0.60
Aspirin	67	53	14	1.00
ACEI or ARB	36	26	10	0.17
Diuretics	27	19	8	0.24
Lipid lowering agents	64	50	14	0.72
Insulin	11	7	4	0.22
Oral antidiabetics	8	3	5	< 0.01
Diabetes	16	9	7	< 0.02
Hypertension	26	19	7	0.38
Atrial fibrillation	3	2	1	0.50
Current smoker	3	3	0	1.00
Myocardial infarction	47	38	9	0.78
CABG	57	44	13	0.54
PCI	45	37	8	0.57
CABG or PCI	66	51	15	0.44
Pacemaker	6	3	3	0.10
CCS IV	12	10	2	0.46
CCS III	58	47	11	
CCS II	8	5	3	
CCS I	0	0	0	
Years with CAD	11.5, 1–35	11.5, 1–35	11, 2–33	0.98
EF > 50%	48	39	9	0.76
EF 50 – 41%	14	10	4	
EF 40 – 30%	14	11	3	
EF < 30%	2	2	0	

EECP = enhanced external counterpulsation, ACEI = angiotensin converting enzyme inhibitor, ARB = angiotensin type I receptor blocker, CABG = coronary artery bypass graft, PCI = percutaneous coronary intervention, CCS = Canadian Cardiovascular Society classification, CAD = coronary artery disease, EF = ejection fraction.

Informed written consent was obtained from all patients included in the study. The study was conducted in accordance with the declaration of Helsinki and ethical guidelines determined by the ethics council at Lund University. Patients who were forced to leave the follow-up due to the need of a new course of treatment were considered to have fallen back to the same CCS class as at referral.

### EECP

The EECP device consists of three paired pneumatic cuffs applied to the calves, thighs and buttocks (Vasomedical, Westbury, New York, USA). The cuffs are inflated sequentially, applying 250 – 300 mmHg of external pressure during diastole, causing the return of blood from the legs to the central circulation and producing aortic diastolic augmentation thus increasing both venous return and cardiac output. The cuffs are then rapidly deflated at the end of the diastole (thereby) reducing peripheral resistance and cardiac afterload. Treatment was given as 35 one-hour sessions administered five days a week for seven consecutive weeks.

### Statistics and calculations

All calculations and statistics were performed using GraphpadPrism 4.0. Descriptive data are presented as mean and standard error of the mean (SEM) or median and range depending on whether the material is normally distributed or not. Statistical significance was assumed when *p *< 0.05. Student's t-test was used when comparing two groups and Fisher's exact test or the chi-2-test was used when comparing baseline factors between responders and non-responders. AVOVA with Dunn's post hoc test was used when comparing more than two groups. The risk for mass significance was not taken into consideration when analysing the results.

## Results

### Response to therapy

Response to therapy, defined as improvement by at least one CCS class, was seen in 79% of the patients. Among the responders, 13% improved two CCS classes or more. Failure to respond to therapy occurred in 21% of the patients. Two patients among the non-responders had improved their CCS class at the 6 months follow-up in spite of failure to respond immediately after therapy.

**Figure 1:**
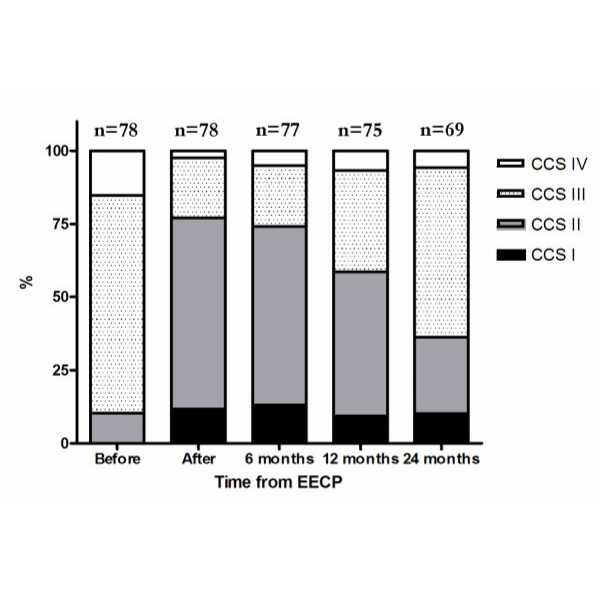
**Overall changes in CCS class before EECP, after EECP and during follow-up**. The figure shows marked reduction in the number of patients suffering from severe angina pectoris after treatment and during the follow-up period.

**Figure 2:**
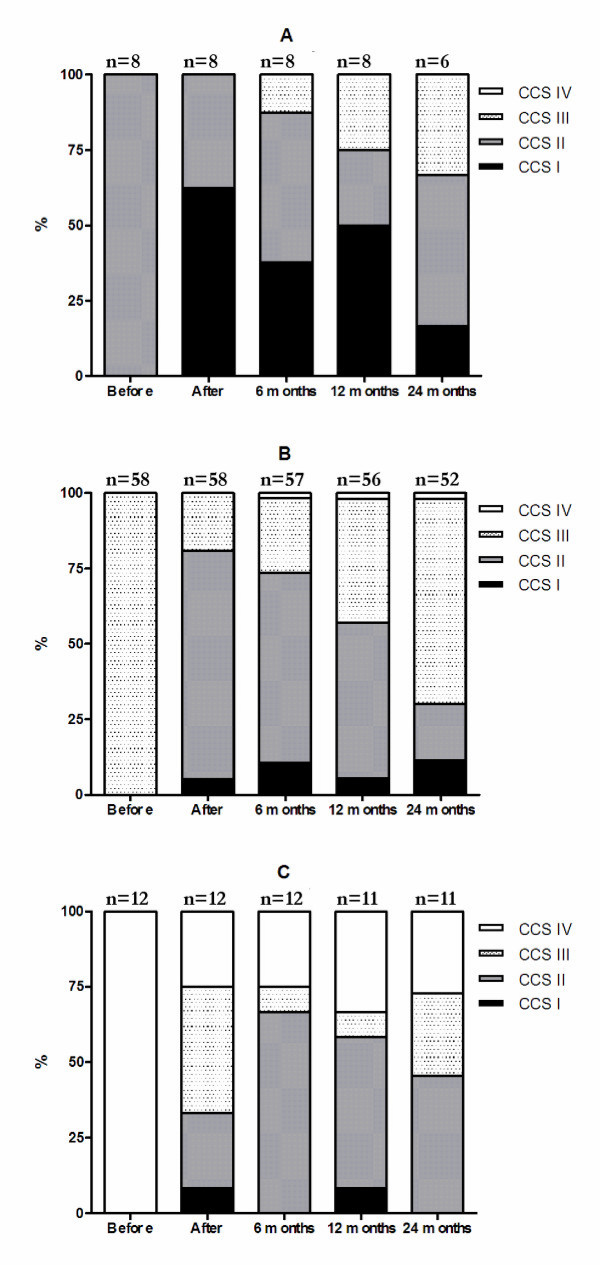
**Changes in CCS class over time**. The figure shows CCS class distribution over time, subdivided into groups depending on CCS class at referral. A = CCS II, B = CCS III and C = CCS IV at referral.

During follow-up, sustained response to therapy was seen for at least 12 months among 61.5% of the responding patients, while 29% of the patients maintained a response to therapy for at least 24 months (*p *< 0.001 and < 0.01 vs. pre-treatment, figure 1). Patients in CCS class III and IV at referral maintained response to therapy for at least 24 months in 22.4% and 70% of the cases, respectively. Patients in CCS class II at referral showed initial response to therapy, but failed to maintain the reduction in anginal status for 24 months (figure 2).

The CCS class improved by, in general, one class after EECP treatment (3.05 before versus 2.14 after treatment).

Weekly GTN usage was reduced in 64% of the patients (*p *< 0.001, Table 2) and none of the patients had to increase their use of short acting nitrates after completing EECP therapy.

**Table 2:** GTN usage before and after treatment

	Before EECP	After EECP
0 administrations/week	9	30
1–2 administrations/week	7	16
3–7 administrations/week	17	19
> 7 administrations/week	44	7
Data missing	1	6

EECP = Enhanced external counterpulsation.

Anginal status did not worsen in any of the patients after EECP treatment.

### Adverse events

Eight patients suffered from adverse events during the EECP therapy and had to terminate their treatment prematurely, including 2 patients who died due to myocardial infarction (Table 3). These patients were excluded from the follow-up investigations. A total of 13 patients had to abort their participation in the follow-up (Table 4). Ten of them left due to recurring angina pectoris in need of further EECP treatment. These patients are included in the study, but calculations were adjusted as if they relapsed to pre-EECP CCS class at the time of abortion. One patient left the follow-up program satisfied with his current anginal status and did not wish to participate in further evaluation. Three patients died during the follow-up. The cause of death remains unknown in the first case while the second patient died from cardiac faliure and the third patient from end-stage amyloidosis.

**Table 3:** Adverse events during treatment

Patients (gender, age)	EECP sessions before termination	Cause of termination
Male, 50	12	Increased chest pain
Male, 84	15	Death due to myocardial infarction
Female, 57	2	Emesis
Male, 58	6	Hiatus hernia
Male, 53	25	Biliary colics
Male, 77	9	Hemorrhoids
Male, 74	25	Chest pain
Male, 59	9	Death due to myocardial infarction

EECP = Enhanced external counterpulsation.

**Table 4:** Adverse events during follow-up

Patients (gender, age)	Months in follow-up before termination	Cause of termination
Male, 52	9	Relapsing angina*, CCS III
Male, 70	11	Withdrawal from follow-up
Male, 64	9	Relapsing angina*, CCS III
Male, 71	10	Relapsing angina*, CCS III
Male, 66	9	Relapsing angina*, CCS IV
Male, 62	8	Relapsing angina*, CCS III
Male, 73	14	Relapsing angina*, CCS III
Male, 53	12	Relapsing angina*, CCS III
Male, 57	10	Relapsing angina*, CCS III
Male, 74	20	Death due to pneumonia and cardiac failure
Male, 71	10	Relapsing angina*, CCS III
Male, 71	4	Death of unknown causes
Female, 79	14	Death due to amyloidosis
Male, 71	21	Relapsing angina*, CCS III

* = Patients with relapsing angina pectoris in need of further therapy during the follow-up. CCS = Canadian Cardiovascular Society classification.

### Analysis of background factors

Diabetes mellitus, use of calcium channel blockers and use of oral antidiabetic agents were each more common among non-responders than among those responding to therapy (*p *< 0.05, *p *< 0.05 and *p *< 0.01, respectively, Table 1). The other baseline factors studied did not affect the outcome of EECP treatment (Table 1).

Smoking and previous myocardial infarction were more common among patients that were forced to withdraw from the follow-up due to recurring angina pectoris (both *p *< 0.05).

## Discussion

The present study is a follow-up study performed on the results of EECP treatment of patients with refractory angina pectoris at a major Scandinavian medical centre for EECP. A majority of the patients were men suffering from long time extensive coronary artery disease refractory to further medical or surgical therapy. This study confirms the safety and efficiency of EECP as a method for reducing CCS class scores in patients with refractory angina pectoris. The reduction in anginal symptoms lasted for up to two years. These results are in accordance with similar studies of long term benefit performed at American medical centres [9-11]. Five patients died during the study, three patients during the follow-up and two during the actual treatment. This is well within what can be expected in this group of patients with end stage coronary artery disease. The overall mortality among a similar set of patients receiving SCS has been shown to be 7–8%, the majority due to cardiac death [12]. Studies where other modes of treatment for refractory angina pectoris have been used report an annual in-treatment mortality of 5–17% [13]

The exact mechanism by which EECP exerts its effect on the cardiovascular system is not fully known. The immediate benefits are similar to those provided by the intra-aortic balloon pump, namely increased blood flow through coronary vessels during diastole and decreased afterload during systole [5]. EECP has been shown to be more efficient than intra-aortic balloon pumping in increasing venous return and enhancing cardiac output [6].

Long term effects of EECP treatment are thought to be mediated through shear stress on the vascular endothelium, which in turn triggers angiogenesis and improves vascular endothelial function [14] due to modulated release of vasoactive substances such as endothelin [15], nitric oxide [15] and vascular endothelial growth factor [16]. Improvement in oxygen uptake after treatment with EECP implies alterations in cellular metabolism as well as hemodynamic improvement [16,17]. EECP has been shown to extend time until exercise-induced ischemia occurs [18]. This may be caused by lowered oxygen demand due to lowered left ventricular afterload and optimization of aortic augmenting index [19]. Relief in myocardial ischemia as well as improved quality of life has been shown in a number of studies [20,21]

Improvement in quality of life may at least in part be due to a placebo effect as described by Springer *et al *[15], but since the current patients suffer from end stage coronary artery disease, are incapacitated and without conventional treatment options, any additional treatment that relieves their pain without adverse effect on their condition is worth taking into consideration. Available, data suggest that EECP improves coronary and systemic perfusion by enhancing function of the vascular endothelium, by favouring angiogenesis and reducing oxygen consumption.

The results from the present study indicate that the outcome of EECP treatment depends on CCS class at referral ("the worse the better"), and negatively on the presence of diabetes mellitus and calcium channel blockers. CCS class II at referral and diabetes [22,23], have previously been described as predisposing to treatment failure. Further studies have to be conducted in order to determine the exact mechanism by which they impair the therapeutic effect of EECP. A possible explanation can however be hypothesized when it comes to calcium channel blockers. Calcium channel blockers act as vasodilators reducing total peripheral resistance. A patient under the influence of calcium channel blockers might fail to mount further vasodilatation, thereby reducing or obliterating the peripheral effect of EECP treatment since dilated blood vessels suffer from less shear stress than occluded ones. Thus, the treatment may be more effective in patients with severe vascular narrowing.

The duration of sustained improvement might be impaired by smoking as seen among those patients who had to cancel their follow-up due to recurring angina pectoris. The explanation could be local effect on the vascular endothelium since tobacco smoking is known to cause endothelial dysfunction [24,25], thereby negating the beneficial effects of shear stress and release of vasoactive factors in the long run.

The present study confirms previous results that EECP is a safe and efficient method to alleviate the symptoms in severe refractory angina. The effects on long time mortality are however yet to be determined. Further studies on background factors associated with reduced response to EECP are needed to confirm the findings presented in this study.

### Limitation of the study

This follow-up report does not include a control group, making the placebo effect a possible confounding factor. The obvious effect of EECP could therefore, at least in part, be the result of increased attention to the patients during treatment and follow-up. Evidence from this and previous studies suggest that EECP treatment is effective in limiting symptoms of severe angina pectoris. No such conclusions can be made of the effect on mortality. Small differences in background factors can be difficult to detect in a material of this size. The possibility of mass significance in the analysis of background factors associated with treatment failure cannot be ruled out since multiple statistical analyses has been performed on the same material. This has to be taken into consideration while evaluating the results of this study. In the results section, some data (average CCS class) is presented as mean when it is ordinal rather than continuous. This is a common approach in biomedical literature, but should be pointed out.

## Conclusion

This study confirms the safety and efficiency of EECP as a treatment option for patients suffering from severe angina pectoris refractory to further pharmacological or surgical intervention. Therapy is most beneficial for those in CCS class III and IV who demonstrated a sustained improvement at the two year follow-up. No such long term response to therapy could be verified among patients with CCS class II angina pectoris at referral. Diabetes mellitus and calcium channel blockers were each significantly more common among patients who failed to respond to EECP. Tobacco use and previous myocardial infarction were associated with early relapse in angina in spite of an initial response.

## Competing interests

The authors declare that they have no competing interests.

## Authors' contributions

TP was responsible for the treatment of the patients and was involved in initiating and designing the study. SB collected most of the data. AE was involved in analyzing the data and writing the manuscript. LE supervised the writing of the final manuscript. All authors have read and approved the final manuscript.

## Pre-publication history

The pre-publication history for this paper can be accessed here:


